# Behavioural change for Parkinson’s Disease: A randomised controlled feasibility study to promote physical activity and exercise adherence among people with Parkinson’s: study protocol

**DOI:** 10.12688/hrbopenres.13843.2

**Published:** 2024-05-29

**Authors:** Leanne Ahern, Suzanne Timmons, Sarah E. Lamb, Ruth McCullagh

**Affiliations:** 1 Discipline of Physiotherapy, School of Clinical Therapies, University College Cork, Cork, Ireland; 2 Centre for Gerontology and Rehabilitation, School of Medicine, College of Medicine and Health, University College Cork, Cork, County Cork, Ireland; 3 Faculty of Health and Life Sciences, University of Exeter, Exeter, England, UK

**Keywords:** Parkinson’s Disease, behavioural change intervention, exercise self-efficacy, exercise adherence, physiotherapy, theoretical domains framework.

## Abstract

**Background:**

Parkinson’s is a common progressive neurological condition characterized by motor and non-motor deficits. Physical activity and exercise can improve health, but many people with Parkinson’s (PwP) have trouble reaching the recommended dosage. Our recent literature review found improvements in exercise adherence with behavioural change interventions, but it remains unclear which are most effective. Further qualitative research and patient and public involvement has informed a novel behavioural change intervention to be tested alongside an existing exercise program.

**Objective:**

To examine the feasibility of behavioural change techniques delivered alongside an exercise programme to improve physical activity, function, and self-efficacy in PwP (and study procedures) to inform a future pilot RCT trial.

**Methods:**

A parallel-arm single blinded randomised feasibility study. Twenty participants with Parkinson’s (Hoehn and Yahr stage 1-3) will be recruited from a physiotherapy primary-care waiting list. Following written consent, and baseline assessment, the participants will be randomly allocated to the intervention (n=10) or the control group (n=10). Both groups will receive usual care, which includes a weekly program of a multidisciplinary education, a supervised exercise class and a prescribed home exercise program. The intervention group will receive additional behavioural change techniques, targeting behaviour regulation, belief about capabilities and social influences. Class and home exercise adherence, behavioural component uptake and adherence, and negative events will be recorded. Outcomes will include enrolment and maintenance rates, physical function, falls, physical activity, and exercise self-efficacy measured pre- and post- the 12-week program (in-person). Surveys will be used to compare experiences and satisfaction between groups. Exit interviews will be completed with the intervention group only, exploring their experience of the behavioural change techniques.

**Discussion:**

The results will help inform a future pilot RCT, based on the intervention acceptability, consent rate, maintenance, and protocol integrity.

**Trial Registration:**

ClincialTrials.gov NCT06192628

## Introduction

Parkinson’s is a progressive neurological condition characterized by both motor and non-motor features. Worsening motor features including balance, postural and gait impairments can lead to a sedentary lifestyle, which deteriorates with disease progression ^1^, contributing to poor quality of life ^2^, loss of functional independence ^2^ and reduced physical condition ^2^. Exercise is important for people with Parkinson’s (PwP) to help control motor and non-motor symptoms ^3^, improving sleep, mood, and memory; it may have a neuroprotective effect, possibly slowing disease progression ^4, 5^. Leisure-time physical activity has been found to reduce motor symptoms, regardless of nigrostriatal degeneration ^6^. Despite this growing evidence, as little as 30% of PwP achieve the World Health Organisation recommended activity levels ^1, 7^, some are inactive for 70% of the day, and most are one-third less active than age-matched peers ^7, 8^.

Ellis *et al.* ^9^ has identified self-efficacy and outcome expectations as strong predictors of exercise adherence. However, PwP have reported that one of the primary reasons for poor participation in regular physical activity is lack of motivation ^10^. Dopamine function, which is associated with motivated behaviour ^11^, is affected in Parkinson’s thus compromising behaviour-reward processing in PwP ^12, 13^. With Parkinson’s progression, there is reduced dopaminergic innervation of the ventral striatum, which is connected to motivational functions ^14^. This may affect PwPs’ well-being, compliance with and participation in exercise programs and inclination to engage in physical activity ^15^. It is thus necessary to develop programs that can motivate people to engage in physical activity.

Such programs, promoting engagement and adherence to physical activity and exercise in PwP, are limited, and few studies have investigated interventions consistent with behavioural change. Speelman *et al.* ^16^ has shown that behavioural techniques (coaching, goal setting, use of activity monitors) are widely accepted by participants, with 90% reporting benefits from the intervention and 73% reporting they would recommend it to other PwP ^16^; the activity monitor being identified as the most useful tool ^16^. The ParkFit trial ^17^, a 2-year, multi-centred, randomized controlled trial comparing physical therapy emphasising behaviour change (ParkFit program), with matched physical therapy emphasizing safety (ParkSafe program), reported no improvement in physical activity, measured by a self-report questionnaire (their primary outcome measure) ^17^. However, both the activity diary and the activity monitor data suggested significantly more physical activity in ParkFit participants compared to the ParkSafe group ^17^. Ellis *et al.* ^18^ implemented a single arm clinical study, with a virtual activity coach to promote daily walking. They showed that encouraging walking, monitoring progression, and overcoming barriers with collaborative problem-solving, was well accepted among participants, and led to improvements in adherence to walking, and improved walking speed ^18^. We recently conducted a systematic review to examine the effects of behavioural change interventions on exercise self-efficacy and adherence in PwP ^19^. We found that behavioural change methods combined with exercise showed no effect on exercise self-efficacy or adherence but brought small improvements in quality of life and physical function compared to exercise alone ^19^. Furthermore, by mapping the data to the Theoretical Domains Framework developed by Atkins *et al*. ^20^ we identified *belief about capabilities*, *goals*, *behaviour regulation*, *social influences* and *reinforcement* as the five most effective domains, which healthcare providers should encompass when developing a behavioural change program ^19^.

Limitations in the available literature includes inadequate sample sizes, with the majority of the studies being non-randomised trials, and limited reporting of the theoretical perspectives, key barriers to implementing the intervention, and rationale for the intervention dose and type of activity ^17, 18^. To date, there is only one definitive multi-centred randomised controlled trial (RCT) ^17^ investigating the effectiveness of a behavioural change intervention on exercise adherence among PwP, highlighting the need for more large-scale RCTs. Furthermore, this RCT ^17^ did not assess exercise self-efficacy, which has been identified as a strong predictor of exercise adherence ^18^. Our recent qualitative research ^21^ displayed difference exercise preferences between men and women (such as setting, type, group vs individual) and motivators to exercise, which we will further examine in this study. Clinicians should develop exercise programs targeting individual’s motivators and perceived needs, thus improving long-term participation ^21^.

The Parkinson’s Exercise and Education Program (PEEP) is an on-going program run in a Primary Care Centre in Cork city. Informed by existing literature, the program was developed by the first author (LA) in 2021 and modified according to physiotherapy colleagues’ and patients’ reviews. The program is targeted for PwP, at Hoehn and Yahr stage 1-3 (i.e., still physically independent). The program runs for 12 weeks and consists of weekly disease self-management education (45-minutes) an exercise component (45-minutes) and a prescribed home exercise plan.

### Research objectives

The aim of this study is to examine the feasibility and acceptability of adding behavioural change techniques to an existing Parkinson’s Exercise and Education Program for people with mild-to-moderate Parkinson’s disease and the study procedures for a future pilot trial. The behavioural change techniques are designed to improve their confidence in managing and progressing their exercise and physical activity independently.

Using mixed methods, the research objectives are:

a) To evaluate the recruitment process and resulting sample characteristics (screen failures, consent refusals).
b) To examine the feasibility of the data collection procedures and outcome measures
c) To explore the acceptability and suitability of the intervention and the study procedures to participants.
d) To assess the time resources required to manage and implement the study and intervention.
e) To explore the trends in outcome measures in terms of range and variability across the entire group.

## Methods

This protocol is reported in line with the SPIRIT 2013 Statement: Defining standard protocol items for clinical trials guidelines ^22, 23^ and the intervention description is guided by the Template for Intervention Description and Replication (TIDieR) ^24^. The protocol is reported according to the Consolidated Standards of Reporting Trials (CONSORT) statement extension for randomised pilot and feasibility studies ^25^. *[Extended Data: SPIRIT Checklist].*

### Patient and Public Involvement (PPI)

We held two one-hour meetings with a PPI group (consisting of 4 PwPs; 3 female and 1 male). In one meeting, the findings of our systematic review and qualitative study were presented, and we gained their input on the draft design for the feasibility intervention. In the second meeting, the PPI group read and provided feedback on the participant information sheet and consent form.

### Study design

A parallel single-blinded feasibility study comparing an existing Parkinson’s Exercise and Education Program (PEEP, control) and an augmented Parkinson’s Exercise and Education Program with behavioural change supports added (PEEP + BC, intervention). Ethical approval has been granted from the local research ethics committee (CREC registration number: ECM 4 (m) 20/06/2023 & ECM 5 (4) 06/07/2023).

### Participants and recruitment

Participants will be recruited from the current waiting list to attend the existing PEEP Program. We plan to recruit 20 participants (10 in each arm) – to optimise numbers while keeping safety paramount in the classes (n=5 per class). We will aim to recruit an equal representation of both sexes (based on self-report). We estimate an attrition rate of 20%, based on previous PEEP programs and available literature ^26, 27^. Prior to random concealed allocation, a member of the research team (LA) will screen participants for eligibility, as follows:

- Diagnosis of idiopathic Parkinson’s Disease, confirmed by neurologist or geriatrician.
- Early Stages of Parkinson’s, as determined by Hoehn & Yahr Stage 1–3 using the United Parkinson’s Disease Rating Scale).
- Ability to drive or obtain transport that will drop and collect at the health centre.
- Ability to independently walk (with or without a walking aid).
- Reported by the referring healthcare provider or carer as able to follow instructions and carry out the exercise program independently at home.

Participants will be excluded if they have:

- A diagnosis of atypical Parkinson’s (e.g., progressive supranuclear palsy, multiple system atrophy, etc) or vascular Parkinsonism or drug-induced parkinsonism
- Previously completed the PEEP program.
- Had a hospital admission < 6 weeks ago.
- Immobility, or are a wheelchair-user.
- Severe visual or auditory impairment
- Serious medical conditions in major organs (heart, lung, or kidney) or other illnesses which prevent independent ambulation or safe exercise.
- Been identified as a high falls risk (identified during the pre-screening using objective measure the Short Physical Performance Battery (a score of ≤6 indicates a higher fall risk in old adults ^28^) and subjective reporting of two or more falls in the past year.

All participants must provide GP clearance letters deeming them medically safe to partake in the exercise component prior to enrolling in the study. Written consent will be obtained from the participants (by LA) before data collection. Participants will be informed that they may withdraw from the study at any time for any reason.

### Randomisation

**Figure 1.**
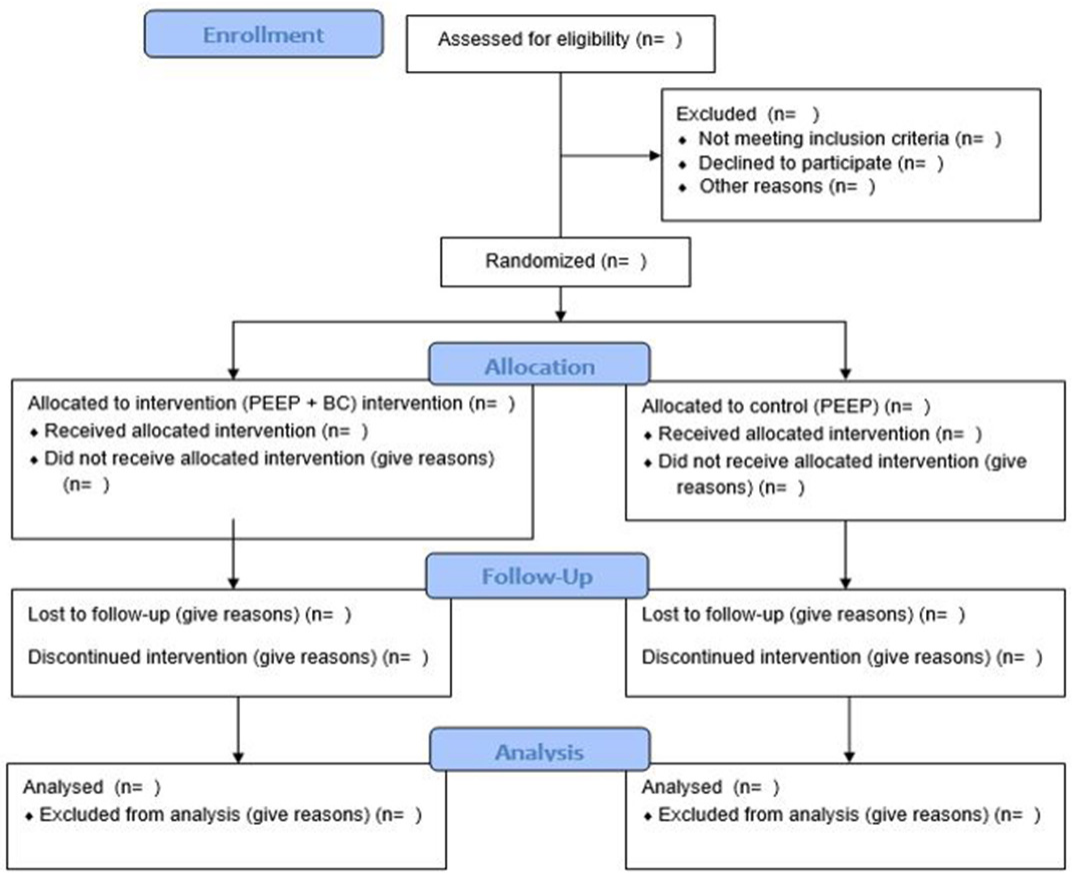
Consort Flow Diagram detailing the participants planned pathway through the trial ^25^.

Following written consent and baseline assessment (to ensure blinded assessment), a research staff member (RMcC), not involved in the screening or data collection, will randomly assign eligible participants in a 1:1 ratio, stratified by sex, using a computer-generated randomisation sequence and concealed allocation (sealed envelopes), to the PEEP or the PEEP + BC group. Outcome assessors will be blinded to group allocation. Study participants will be instructed not to share any information on group allocation with the assessor or with other PwP outside the intervention. Every attempt will be made to ensure the assessor is not unblinded; if this occurs, a substitute assessor will be used. The assessor will not be in the facility while the program is being delivered. A full CONSORT ^25^ flow diagram is presented in Figure 1 and will be populated throughout the study. The participant timeline is documented in Table 1.

**Table 1.** Participant Timeline.

	Enrolment		Allocation	Post Allocation	
Timepoint	-T _2_ _(two weeks before_ _baseline)_	-T _1_ _(Week before_ _baseline)_	0	T _0_ _(baseline)_	T _1 (post-intervention_ _– Week 12)_
**Enrolment**:					
Eligibility Screen	X				
Informed Consent	X				
StepWatch Activity Monitor distributed to patients		X			
Baseline Assessment		X			
Group Allocation			X		
**Intervention:**					
PEEP + BC				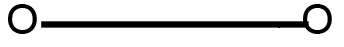	
PEEP				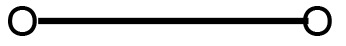	
**Primary Outcome measures:**					
Walking Activity (SAM)				X	X
Gait and Balance (APDM Mobility Lab system™)				X	X
Exercise self-efficacy (ESES)				X	X
**Secondary Outcome measures:**					
Physical Endurance (6MWT)				X	X
Quality of Life (PDQ-39)				X	X
Exit Interviews					X
Safety					X

**SAM**: StepWatch Activity Monitor; **ESES**: Exercise self-efficacy scale; **6MWT**: 6-minute walk test; **PDQ**- **39**: Parkinson’s Disease Questionnare-39 **Safety**: Falls, hospitalisation, muscle soreness

### Usual care and intervention details

Materials include gym and exercise equipment [chairs, steps, resistance bands, ankle weights, water bottles (will be used as hand weights)], an Exercise Diary [ *Extended Data: Appendix A]* and a Behavioural Change Template [ *Extended Data: Appendix B*]. One registered physiotherapist (LA) with a special interest in Parkinson’s Disease will be leading the programme, as part of their doctorate training. A physiotherapy assistant, with additional training as a Postural Stability Instructor will be present at all the exercise sessions to ensure safe, correct exercise completion, which will allow the physiotherapist to focus on the behavioural change techniques.

***Usual Care: Parkinson’s Exercise and Education Program (PEEP).*** The usual care consists of three components:

(1) Weekly group exercise class. Classes will be delivered in a primary care physiotherapy gym. They will last approximately 45 minutes, including a five-minute warm-up and cool-down. The exercises class will include eight different exercises (focusing on strength, dynamic balance, coordination, flexibility, and dual tasking). The exercise type will remain consistent throughout the 12 weeks (to aid memory retention), however the progression of exercises will be tailored to each participant’s capability, weekly, by increasing the number of repetitions and step height, heavier resistance bands, addition of hand and ankle weights and adding a cognitive element while performing the exercises. Thus, adjustments in intensity or complexity will be tailored to the individual and progressed with time. Participants will be asked to attend for one hour, to allow 15 minutes for people to settle in, and give an opportunity to socially engage before the exercise class begins. During this time, the physiotherapist (LA) will have a brief 1:1 conversation with each participant about adverse events that may have occurred that week (see section *Safety).*

(2) Twelve weekly 45-minute group education sessions will be delivered by a range of healthcare professionals and guest speakers including a physiotherapist, speech and language therapist, an occupational therapist, dietician, geriatrician, Parkinson’s specialist nurse, psychologist, social prescriber, and social worker.

These sessions will be delivered in a conference room located next to the Physiotherapy gym in the primary care centre and will include general information to signpost people to services, the role of the healthcare profession in question. Education is provided on medication, symptoms, diet, and aids, but information that could improve exercise self-efficacy and self-management of exercise programs (such as rest, pacing, goal setting etc) is not provided. Following each education session, a hardcopy of the lecture presentation will be provided, and any pamphlets and leaflets recommended by the speaker. The education sessions will be delivered to both groups together, on the day of the usual care group’s exercise classes (the intervention group will have their exercise class on a separate day). All efforts will be made to keep contamination to a minimum as the participants will sit with their own group and will be asked not to discuss the exercise classes with others, and staff will be monitoring conversations in the room. Family-members will also be encouraged to attend the education sessions.

(3) At home, the participants will be expected to independently complete the exercises twice weekly, and this will be discussed at each exercise class with the registered physiotherapist.

***Intervention: Parkinson’s Exercise and Education Program with the Behavioural Change Techniques (PEEP + BC).*** The BC intervention will consist of usual care and four additional techniques to address behavioural regulation, belief about capabilities and peer support.

(1) During the weekly exercise class, the intended functional benefit of the exercises will be explained to everyone. Support will be given to self-select personal exercise intensity and to manage their own exercise and progressions at home. Peer support, competition, reward, and fun will be strongly encouraged and supported by the registered physiotherapist and physiotherapy assistant. By week six, the participants will be invited to lead the class, with the class leader rotating each week, aiming to be independently managing the class by week 12.

(2) An exercise diary to be completed independently at home daily [ *Extended Data: Appendix A*]. Details will include the type of exercise or physical activity of choice, and its duration and intensity (using Borg Rating of Perceived Exertion Scale). Furthermore, they will be asked to reflect on the exercise; their confidence/skill completing the activity, consequences of the activity (both positive and negative) and reasons for non-completion. Daily diary completion will take less than 5 minutes.

(3) Individual one-to-one weekly conversations between the registered physiotherapist and the participant, covering the domains of behavioural regulation, peer support and belief about capabilities. The conversations will be held before each weekly exercise class, when the participants are coming into the gym, while others are settling into the room. All topics will be covered frequently but not weekly, to avoid repetition [ *Extended Data: Appendix B*]. The conversation will last two to five minutes per person (approximately 15 minutes in total per class). During this time, the participant’s exercise diary will be reviewed with the physiotherapist to support the participant to explicitly reflect on progress and their expectations, to identify solutions to any barriers, and to set activity goals for the following week.

(4) Peer support will be further supported with the suggestion of a WhatsApp group. To encourage self-management and to limit the influence of sharing their personal information, the physiotherapist will suggest this at the first visit, when, if possible, a participant will volunteer to lead the WhatsApp group. From this point onwards, the physiotherapist will remain uninvolved but will ask about the use of group chat in the individual conversation.

All participants will be asked to continue their usual exercise during the study period.

### Outcomes measures

***Feasibility outcomes.*** Feasibility outcomes of interest include methodological, procedural and clinical uncertainites ^25, 29, 30^, examination of recruitment rates, consequences of eligibility criteria, attrition once recruited, data collection completion, resources needed to complete the intervention and the data collection, participant adherence to the intervention, and acceptability of the intervention and study procedures to participants. Feasibility outcomes are shown in Table 2 ^31^.

**Table 2.** Overview of feasibility outcomes ^31^.

Outcome	Evaluation
Recruitment and eligibility	- Number of patients on current waitlist - Percentage assessed for eligibility; fulfilling inclusion criteria, and included (of total number identified) - Ambiguities regarding eligibility criteria - Reasons for ineligibility - Reasons for non-participation
Data Collection	- Percentage of completed assessments. - Ease of completion and number of missing items - Types and number of potential clinical uncertainties in interviews
Attrition	- Rate of study dropout - Rate of intervention dropout
Resources needed to complete the study and intervention	- Length of time required for: - Participants to work through intervention. - Participants to complete questionnaires and interviews - Study personnel to administer the study
Adherence to the intervention	Number of: - Completed activity diaries. - Exercise classes attended. - Education sessions attended.
Acceptability and experience of the intervention and data collection and exploration of mechanisms of impact	- Reasons for poor attendance and withdrawal from the study and intervention - experience and perception of the behavioural change intervention (including positive and negative consequences) - perceived value of completing home exercise diaries - perceived value of WhatsApp group - perceived value of one-to-one conversations at each exercise class - perceived confidence in self- management - Number and severity of negative events

***Process outcomes.*** The following will be collected to indicate the study process feasibility:

1. Fidelity of delivery of exercise-only and exercise-behaviour change classes (duration; range of exercises included progressing intensity as per plan, etc)
2. Class attendance; drop-out rates; set-up administrative time required (participant queries between classes, room preparation, etc)
3. Completion of home-based (self-reported) exercise activity (exercise type, number of repetitions, intensity)
4. Fidelity and barriers of delivery of the behaviour change intervention (review of the records of one-to-one discussions [ *Extended Data: Appendix B*] and researcher completion of a reflective log after each exercise session.

***Acceptability.*** The acceptability of the class and independent exercise (both groups) will be explored by surveys [ *Extended Data: Appendix C*], and the behavioural component will be explored through additional exit interviews, using a semi-structured interview, with the intervention group only [ *Extended Data: Appendix* D].

***Safety.*** Falls (number and frequency), injuries (cuts, bruises, muscle pains, other injuries), ED attendance or hospitalisation, and death will be considered as adverse events and will be discussed and recorded during the weekly conversations held before each exercise class. If a participant does not attend (without prior explanation) they will be contacted. Family members will be contacted if the participant does not answer over one week, for those who do not have family, the GP will be contacted. Any serious adverse events will be reported to the Chief Medical Officer of UCC.

***Performance outcomes.*** For performance outcomes see Table 1. We will complete all mobility-related assessments during the participants’ ON phase for consistency (i.e., 1–2 hours post medications, and stated “good time of day”)

***Walking activity and falls events.*** The Stepwatch Activity Monitor will be used to measure step-count and physical activity. This is a validated accelerometer, with excellent accuracy in PwP ^32^. The device is ankle-worn and is fully waterproof, allowing seven days of uninterrupted data collection. Participants will be asked to wear the accelerometer for the week before the programme begin, and again, for the final week of the programme. Within-person differences in average daily step count will be used to examine changes in physical activity. As it is well known that activity diaries are unreliable measures of activity ^33^, we will focus on accelerometery data only as a measure of physical/walking activity.

Falls and ‘near falls’ are the only measures that will be taken from the Activity Log. These events will be discussed in both groups during weekly one-to-one discussions about adverse event with all participants (at the beginning of the exercise class) and recorded.

***Gait and balance.*** Physical performance will be measured objectively before and immediately after the program using the APDM Mobility Lab system™ ( http://www.apdm.com/mobility/). The APDM Lab system uses coordinated, wearable inertial sensors to precisely analyse quality of gait and balance ^34^. It is a portable gait and balance laboratory and allows streamlined gait and balance assessment by making it easy to collect, store, analyse, and interpret balance and gait data from a large set of prescribed tasks ^34^. It is a validated device to objectively measure walking and balance in PwP ^34^. Five Opals™ (accelerometers) will be attached to participants two feet (ankles) and two arms (wrists) and one at the waist using Velcro straps (provided). The participants will be asked to stand, walk a 3-metre distance, turn around, walk back to the chair, and sit down (Timed Up and Go Test (TUG)). A 2-minute free walk (at the participants self-selected pace) will be recorded also to examine stride length and turns specifically.

***Exercise self-efficacy.*** Exercise self-efficacy will be measured using the Exercise Self-Efficacy Scale ^35^ (ESES) at baseline, immediately post-intervention. The ESES is a self-report instrument, validated in PwP ^34^, containing ten statements about how confident the person is performing physical activity and exercise ^36^. Responses are chosen from a four-point scale with 1 = not at all true to 4 = always true (score range 10–40, higher indicating more confidence ^36^.)

***Secondary outcome measures.*** We defined two secondary measures:

*Physical endurance*

Physical endurance will be measured with the six-minute walk test ^35^ (6MWT) at baseline and post-intervention. Participants walk continuously for six minutes at their self-selected pace. As recommended, an indoor hallway with the 10m distance will be used, and total distance will be recorded.

*Quality of life (QoL)*

QoL will be measured using the Parkinson’s Disease Questionnaire ^37^ (PDQ-39) at baseline, and post-intervention. The PDQ-39 assesses Parkinson’s disease-specific health-related QoL in the previous month across eight dimensions of daily living ^37^, with specific dimensions of functioning and wellbeing ^37^.

### Data collection

Baseline data will include personal information such as demographics (including age and self-reported sex), social history, medical history, disease severity (using the United Parkinson’s Disease Rating Scale – motor exam) and medication history will be collected before randomisation by the research physiotherapist (LA) and the Stepwatch Activity Monitor will be programmed and, as per the manufacturer’s instructions, attached to the participants ankle at the end of the assessment. They will be instructed to wear the accelerometer continuously for seven days, until they return the following week, when the device is removed the data will be downloaded onto a password protected file on the research laptop. Participants will be encouraged to contact the research physiotherapist with any concerns about the accelerometer during the week, and a courtesy call will be made during the week to ensure data collection.

Similarly, the participants will be asked to wear the accelerometer for the final week of the program. On the last day of the program, the accelerometer will be removed, and the data will be downloaded. The outcome assessments will be completed by a blinded registered physiotherapist, who is fully trained in the completion of the assessments.

***Adherence.*** Full adherence to the intervention will be defined as follows: 1) attending the baseline and immediate post-intervention assessments, 2) completing 12 weekly activity diaries and 3) attending all 12 exercises classes, attending all 12 education sessions. Attendance at the exercise classes and education sessions will be documented each week by the registered physiotherapist. Activity diaries will be viewed each week during the weekly one to one weekly conversations between the registered physiotherapist and the participant before each exercise class.

***Qualitative process evaluation.*** All participants will be asked to complete a feedback survey [ *Extended Data: Appendix C*] with open-ended questions exploring what they liked/disliked about the PEEP program, elements of the program they felt were not required, or elements they believe should have been included in the program, and their opinions of the topics covered during the education sessions. On-line semi-structured interviews exploring the acceptability of the behavioural change techniques will be conducted a research physiotherapist (RMcC) with the intervention participants only, immediately post-intervention (12 weeks). Participants will be asked to discuss the perceived impact and value, and how the intervention address barriers to exercise and physical activity. The interview guide will be informed by previous research examining the acceptability of BC interventions ^38– 40^ coded under the domains of the Theoretical Domains Framework ^20^. [ *Extended Data: Appendix D*]

### Data analysis

***Quantitative analysis.*** With small numbers in each group, we will present and treat all continuous data as non-parametric data. Suitable summary statistics (e.g., median, interquartile range, and frequency, percentages) will be provided for all outcomes.

These summary statistics will be used to assess the feasibility of the intervention. Potential ambiguities regarding standard safety procedures, types and numbers of measures undertaken to assure patient safety, and types and numbers of unforeseen safety issues will be reported and disaggregated by sex. We will analyse the results according to a modified intention-to-treat principle, whereby we will exclude only patients with no follow-up measurements at all.

***Qualitative analysis.*** Data from the semi-structured interview questions will be audio- recorded, transcribed verbatim, and analysed with the data from the surveys using qualitative content analysis. Difference between the sexes will be carefully considered.

## Dissemination of data

A paper describing the main outcomes of the feasibility study, including the qualitative data, will be submitted to a peer review journals and the results will be presented both locally to physiotherapy clinical colleagues, and at international conferences and will be presented to the Cork Branch of the Parkinson’s Association of Ireland. A poster summarising the results in simple terms will be produced and placed on the wall of the Primary care centre.

## Amendments

The publication of the study’s result will include any amendments to this protocol in tabular format including the date, description, and rationale of each amendment (section “Differences between protocol and study”). The clinicaltrials.gov register will remain updated with the protocol and any amendments made.

## Ethical application

Full ethical approval has been granted for the study by Clinical Research Ethics Committee of the Cork Teaching Hospitals (Registration number: ECM 4 (m) 20/06/2023 & ECM 5 (4) 06/07/2023).

## Study status

The feasibility study is due to commence participant recruitment in November 2023.

## Discussion

This intervention has been informed from our previous systematic review ^19^ and qualitative studies ^21^ with participants and healthcare providers. These studies showed us that people want to exercise but are frustrated by the barriers and reliance on healthcare providers or family to manage or motivate them to exercise. We propose to examine the feasibility of a behaviour change intervention to address behaviour regulation, belief about capabilities and peer support by encouraging self-progression, reflection, competition, fun and reward to drive adherence and self-management of exercise behaviours.

The results of this feasibility study will inform a future pilot trial, by examining responsiveness of outcome measures, consent rate, and qualitatively exploring acceptability and experiences of the intervention which will help to refine the intervention and may explain uptake and adherence rates. We will consider sex differences, if any that emerge from the qualitative interviews.

The effects of behavioural change intervention on exercise adherence and exercise self-efficacy remains unclear. There is a clear need for participatory research to design interventions that help PwP to maintain health and wellbeing. While not definitive, this study will help further behavioural change intervention design informed by the published literature, stakeholder input and PPI involvement.

## Data Availability

No data are associated with this article. Dryad: Extended Data for Behavioural change for Parkinson’s Disease: A randomised controlled feasibility study to promote physical activity and exercise adherence among people with Parkinson’s: study protocol, https://doi.org/10.5061/dryad.prr4xgxt5 ^41^. Zenodo: SPIRIT checklist for ‘Behavioural change for Parkinson’s Disease: A randomised controlled feasibility study to promote physical activity and exercise adherence among people with Parkinson’s: study protocol’, https://doi.org/10.5281/zenodo.10407626 ^42^.
