# Supplementary material for: Behavioural change for Parkinson’s Disease: A randomised controlled feasibility study to promote physical activity and exercise adherence among people with Parkinson’s: study protocol
Source: HRB Open Res. 2024 May 29;7:7. Originally published 2024 Feb 16. [Version 2] doi: 10.12688/hrbopenres.13843.2 (PMC11109530; doi:10.12688/hrbopenres.13843.2)
Supplement: Peer review history

# Reviewer report: Reviewer response for version 2

**DOI:** 10.21956/hrbopenres.15253.r44011

**Published:** 2024-12-18

**Authors:** Mark Manago

**Affiliations:** 1 University of Colorado Denver Anschutz Medical Campus, Aurora, Colorado, USA

This is an interesting study protocol designed to increase exercise adherence in PwP. It is overall well-designed to test the primary feasibility aims. The study is well-justified in context of the current literature and preliminary work. I commend the authors for beginning this line of work with a mixed-methods feasibility design and clearly defined feasibility criteria. The active ingredients of the BC intervention are well-justified and clearly described. The report is overall easy to follow and clearly written. However, there are several minor typos that could be addressed by a careful read through.

 Abstract

 In the Objective statement, it is unclear what is meant by “(and study procedure)”. That is, why is this enclosed in parentheses?

 Methods

 What is the justification of having equal representation of sexes when PD typically affects more men? Especially when the PPI group was not equally divided by sex (3F 1M).

 Why will the researchers collect an instrumented 2-minute free walk and then separately collect a 6-minute walk test? It seems unnecessary and fatiguing to do both. Why not instrument the 6MWT or just make the 2-minute walk the test of walking endurance?

 Data Analysis

 I am not sure limiting your analysis to non-parametric statistics is the best option, especially before reviewing distributions. You will potentially get more meaningful/useful data from understanding the variability of the data through standard deviations/ confidence intervals, especially for key outcomes that you might considering powering the next phase on. Maybe consider tempering this language to say something about how you will consider the data distribution during analysis.

 Dissemination

 Could you also include dissemination to patient groups both regionally and nationally?

Is the study design appropriate for the research question?

Yes

Is the rationale for, and objectives of, the study clearly described?

Yes

Are sufficient details of the methods provided to allow replication by others?

Yes

Are the datasets clearly presented in a useable and accessible format?

Not applicable

Reviewer Expertise:

Rehabilitation research in people with MS and PD

I confirm that I have read this submission and believe that I have an appropriate level of expertise to confirm that it is of an acceptable scientific standard.

# Reviewer report: Reviewer response for version 1

**DOI:** 10.21956/hrbopenres.15168.r38320

**Published:** 2024-05-14

**Authors:** Chris Seenan

**Affiliations:** 1 Glasgow Caledonian University, Glasgow, Scotland, UK

The article details a protocol for a randomized feasibility trial comparing standard care to additional behavior change intervention in people with Parkinson's disease. Feasibility and acceptability of the novel approach will be explored recruiting participants from an existing exercise and education class. 

 The objectives and planned outcomes from the study are well matched and described. The protocol is presented according to appropriate reporting guidelines and the additional material provides adequate information that would enable replication. 

 Overall, this is a well-presented protocol describing a useful and well-designed study in an important area of practice. The findings of the study will enable progression within the field and for people with Parkinson's disease.

Is the study design appropriate for the research question?

Yes

Is the rationale for, and objectives of, the study clearly described?

Yes

Are sufficient details of the methods provided to allow replication by others?

Yes

Are the datasets clearly presented in a useable and accessible format?

Yes

Reviewer Expertise:

My expertise is within cardiovascular disease, physiotherapy and developing behaviour change interventions for long term conditions. I have experience designing and implementing feasibility trials in clinical practice.

I confirm that I have read this submission and believe that I have an appropriate level of expertise to confirm that it is of an acceptable scientific standard.

# Response

**Published:** 2024-05-17

**Authors:** Leanne Ahern

**Affiliations:** University College Cork, Ireland

Thank you very much for taking the time to review the study protocol. We greatly appreciate your feedback

# Reviewer report: Reviewer response for version 1

**DOI:** 10.21956/hrbopenres.15168.r38949

**Published:** 2024-05-08

**Authors:** Hanna Johansson

**Affiliations:** 1 Department of Neurobiology, Care Sciences and Society, Karolinska Institutet, Stockholm, Sweden

The authors here describe a study protocol for a parallel-arm single blinded randomised feasibility study involving people with mild-to-moderate Parkinson’s disease. Both groups will receive multidisciplinary education, a supervised exercise class and a home exercise program, but additional behavioural change techniques will be added to the intervention group. Exercise studies, and especially those involving elements of behavioural change, are highly needed in the Parkinson’s research. Evaluating the feasibility of a study design before commencing with a larger scale RCT is of utmost importance, and I commend the authors for doing this valuable ground-work.  

 Below are some questions and suggestions for the authors to consider and address as they find appropriate.

***Feasibility/methodology***

- Consider formulating progression criteria to better help you assess whether and how to progress to a full-scale RCT (see Mellor K, et.al.,2023 [Ref 1] for recommendations.)
- Considering that the authors plan to perform a RCT on a smaller scale, and to assess most elements of this process, I would say that this is in fact a pilot RCT. How come the authors do not intend to use the results of this study to inform a larger scale RCT, instead of planning to perform yet another pilot study?
- Please consider evaluating the randomization process as well as the ability to maintain blinding throughout the study.
- Will the results of this study help inform the choice of a primary outcome for the planned RCT? If so, please describe how.

***Participants/recruitment/screening***

- How will a diagnosis of idiopathic PD be confirmed?
- How will Hoehn and Yahr stage be confirmed? Using the MDS-UPDRS III?
- Fall risk and fall history will be assessed during pre-screening. Please describe the cut-off used to define “frequent falls”.
- Have the authors considered excluding participants who are already highly physically active in their daily life? It is a common problem in exercise studies that people who are already active tend to sign up, thereby resulting in biased samples and lowered ability to show effect. If physically active people are recruited, please make sure participants are willing to continue with their usual exercise during the intervention period.

***Outcomes/assessments***

- Have the authors considered evaluating cognitive function?
- Have the authors considered evaluating symptoms of depression or anxiety since this can negatively affect exercise adherence/motivation etc?
- Why not use accelerometers the week after the intervention has ended, instead of letting participants wear them the last week of the intervention?
- Perhaps just a typo, but I can only see two secondary outcome measures (6-min walk test and PDQ-39) even though the authors state that there should be four.
- What is the rationale for performing both the 2-minute walk test and the 6-minute walk test? Will both tests be performed at self-selected (i.e., usual) pace?
- The authors state that the recommendation for the 6-minute walk test is to conduct it in an indoor hallway over a 10-meter distance, but this is not correct. The American Thoracic Society recommends it be performed over 100 ft (apx 30 meter). If possible, I therefore suggest that both the 2- and the 6-minute walk test be performed over a 30 meter distance.
- The TUG test is not described correctly (does not describe the first turn and the 3 meter walk back to the chair).
- Why use five and not six opals? Please consider adding the sternum opal as well so that you can assess sit-to-stand/stand-to-sit transfers during the TUG.

***Intervention***

- Regarding the home exercise diary, I am curious as to how difficulty level will be explained to participants. Is this based on physical exhaustion or other?  If so, have you considered letting participants rate this using for example Borg RPE instead?
- The behavioural change template (Appendix B) seems a bit too exhaustive to complete in 2-5minutes. The authors state that not all questions will be addressed each week, but how will you choose which ones to focus on each week?
- I think the authors have done a great job of describing both the control group and the intervention group, but I wonder whether it would be good to have a table outlining all elements as well to get a better overview of which elements are overlapping versus those that are group-specific.

Is the study design appropriate for the research question?

Yes

Is the rationale for, and objectives of, the study clearly described?

Yes

Are sufficient details of the methods provided to allow replication by others?

Yes

Are the datasets clearly presented in a useable and accessible format?

Not applicable

Reviewer Expertise:

Parkinsons disease

I confirm that I have read this submission and believe that I have an appropriate level of expertise to confirm that it is of an acceptable scientific standard.

## References

[1] : Recommendations for progression criteria during external randomised pilot trial design, conduct, analysis and reporting. *Pilot Feasibility Stud* .2023;9(1) : 10.1186/s40814-023-01291-5 59 10.1186/s40814-023-01291-5 37061720 PMC10105402

# Response

**Published:** 2024-05-17

**Authors:** Leanne Ahern

**Affiliations:** University College Cork, Ireland

Thank you very much for taking the time to review the study protocol. We appreciate your suggestions, many of which we have added, and we hope that we have addressed them appropriately. We have detailed the responses below.

***Feasibility/methodology***

- Consider formulating progression criteria to better help you assess whether and how to progress to a full-scale RCT (see Mellor K, et.al.,2023 [Ref 1] for recommendations.)

***Response: **Thank you for your suggestion – it will be valuable step in our future pilot study.*

- Considering that the authors plan to perform a RCT on a smaller scale, and to assess most elements of this process, I would say that this is in fact a pilot RCT. How come the authors do not intend to use the results of this study to inform a larger scale RCT, instead of planning to perform yet another pilot study?

***Response: **Thank you for your query. As a team, we considered this quite extensively, and decided that there were too many unknowns for this to be a pilot study. To tease apart the different components was an important aim of the study, rather than to consider the intervention as one multicomponent intervention. Therefore, we decided to test the intervention as a feasibility study, allowing us to take the intervention apart, as such, and use the qualitative feedback to further inform our decisions.  *

- Please consider evaluating the randomization process as well as the ability to maintain blinding throughout the study.

***Response: **Thank you for highlighting this. We agree that the methods can lead to bias. Unfortunately, for this study with existing resources, we are unable to change these, but will be considered for future studies, including the planned pilot.  *

- Will the results of this study help inform the choice of a primary outcome for the planned RCT? If so, please describe how.

***Response: **We anticipate that the intervention may be further refined from this study. We plan to analyse for trends of change, and to examine which measures appear to be more sensitive to change, however, we do not anticipate that we will choose the primary outcome measure from this study. Our current focus is more on feasibility of data collection. * 

***Participants/recruitment/screening***

- How will a diagnosis of idiopathic PD be confirmed?

***Response: **Thank you for bringing this to our attention. Idiopathic PD will be confirmed by a consultant neurologist or geriatrician. This is part of the referral process. We have added this in the text, under Participants and recruitment; Page 6, line 6.*

- How will Hoehn and Yahr stage be confirmed? Using the MDS-UPDRS III?

***Response: **Yes, that is correct – we have added this detail. - Page 6, line 7.*

- Fall risk and fall history will be assessed during pre-screening. Please describe the cut-off used to define “frequent falls”.

***Response: ** We defined frequent falls as two or more falls in the last year, based on Goodwin et al. ^1^ and have added this to the exclusion criteria in the text - Page 6, line 23.*

- Have the authors considered excluding participants who are already highly physically active in their daily life? It is a common problem in exercise studies that people who are already active tend to sign up, thereby resulting in biased samples and lowered ability to show effect. If physically active people are recruited, please make sure participants are willing to continue with their usual exercise during the intervention period.

***Response: **Thank you for your suggestion. The main aim of this study was to test the feasibility of the data collection and the programme delivery. We are limited by time and resources. We will use this as an for the future RCT.  We have added in the text that we will ask all participants to continue with their usual exercise during the intervention period – page 10, line 24*

***Outcomes/assessments***

- Have the authors considered evaluating cognitive function?

 
***Response: **We have not considered it for this feasibility study, but this will be considered for the future pilot study and RCT*

- Have the authors considered evaluating symptoms of depression or anxiety since this can negatively affect exercise adherence/motivation etc?

***Response: **Again, we have not considered it for this pilot, but going forward, we can see its value in future studies.*

- Why not use accelerometers the week after the intervention has ended, instead of letting participants wear them the last week of the intervention?

***Response: **We selected the last week of the intervention as we would be able to collect them easily from the participants at the last session, rather than ask them to return / send the accelerometers back to us. For earlier studies, we have experience of them being misplaced or lost. Many forget they are wearing them.*

- Perhaps just a typo, but I can only see two secondary outcome measures (6-min walk test and PDQ-39) even though the authors state that there should be four.

***Response: **Thank you for bringing this to our attention. We have corrected this, page 10, line 18.*

- What is the rationale for performing both the 2-minute walk test and the 6-minute walk test? Will both tests be performed at self-selected (i.e., usual) pace?

***Response: **We will perform the 2-minute walk test using the APDM data collection. The 6MWT will be performed separately to measure physical endurance. Yes, both are performed at self-selected pace. The report has been reworded to clarify this.*

- The authors state that the recommendation for the 6-minute walk test is to conduct it in an indoor hallway over a 10-meter distance, but this is not correct. The American Thoracic Society recommends it be performed over 100 ft (apx 30 meter). If possible, I therefore suggest that both the 2- and the 6-minute walk test be performed over a 30 meter distance.

***Response: **Thank you for this important information. We have begun data collection and are now delivering the programme. This will be recognised as a limitation of the study in the final report. In future studies, we will use a 30 m walkway, but will continue to use the 10m walkway to standardize among participants.*

- The TUG test is not described correctly (does not describe the first turn and the 3 meter walk back to the chair).

***Response: **Thank you for bringing this to our attention. We have corrected this, page 10, line 7*

  

- Why use five and not six opals? Please consider adding the sternum opal as well so that you can assess sit-to-stand/stand-to-sit transfers during the TUG.

***Response: **Thank you for your suggestion. We aim to keep the data collection as simple as possible. We decided this protocol based on the advice we received from colleagues in KU Leuven, who advised to collect the data using 5 opals.  Based on our results, we may add the additional sixth opal for future studies.*

***Intervention***

- Regarding the home exercise diary, I am curious as to how difficulty level will be explained to participants. Is this based on physical exhaustion or other?  If so, have you considered letting participants rate this using for example Borg RPE instead?

***Response: **Thank you for your suggestion, we can see the value of this and have added it to the protocol, Page 10, line 5*

- The behavioural change template (Appendix B) seems a bit too exhaustive to complete in 2-5minutes. The authors state that not all questions will be addressed each week, but how will you choose which ones to focus on each week?

***Response: **Thank you for your suggestion. We had planned on using sections of the template each week; not to use the complete template every week. We have divided this into weekly templates and have added these to the repository.*

 References

 1.           Goodwin VA, Richards SH, Henley W, Ewings P, Taylor AH, Campbell JL. An exercise intervention to prevent falls in people with Parkinson's disease: a pragmatic randomised controlled trial. *Journal of Neurology, Neurosurgery & Psychiatry*. 2011;82(11):1232-1238.
